# 二线不同化疗方案治疗小细胞肺癌的疗效和安全性比较

**DOI:** 10.3779/j.issn.1009-3419.2015.05.05

**Published:** 2015-05-20

**Authors:** 治桦 李, 晓晴 刘, 俭杰 李, 红军 高, 传昊 汤, 晓燕 李, 万峰 郭, 海峰 秦, 伟霞 王, 莉莉 曲, 健 陈

**Affiliations:** 1 100071 北京，解放军第307医院肺部肿瘤内科 Department of Lung Cancer, Affiliated Hospital of Academy of Military Medical Sciences, Beijing 100071, China; 2 100088 北京，解放军第二炮兵总医院肿瘤科 Department of Oncology, General Hospital of The Second Artillery of PLA, Beijing 100088, China

**Keywords:** 肺肿瘤, 二线化疗, 缓解率, 生存, 安全性, Lung neoplasms, Second-line chemotherapy, Response Rate, Survival, Safety

## Abstract

**背景与目的:**

小细胞肺癌是一种侵袭性非常强的肿瘤，其主要治疗方案是细胞毒化疗，尽管有较高的初始治疗缓解率，但大部分患者在一线治疗后会出现复发或进展。目前只有较少的证据证明二线治疗能给复发或晚期小细胞肺癌患者带来生存获益，指南推荐药物较多，但临床多依据经验制定方案。本研究回顾性分析小细胞肺癌患者不同二线治疗方案的疗效和安全性，以指导临床医生更客观地选择小细胞肺癌二线治疗方案。

**方法:**

回顾性分析了309例接受二线治疗的小细胞肺癌患者，其中157例患者进展后仅予最佳支持治疗，其余152例患者进行了二线化疗。采用*Kaplan*-*Meier*法生存曲线及*Log*-*rank*检验等统计学方法，观察终点为客观缓解率（objective response rate, ORR）、疾病控制率（disease control rate, DCR）、无进展生存时间（progression-free survival, PFS）、总生存时间（overall survival, OS）和安全性分析。

**结果:**

接受二线化疗的患者较二线仅接受最佳支持治疗的患者生存获益明显，两组患者自一线治疗开始的OS分别为11.5个月和6.0个月（*P* < 0.001），并且前者无论何种复发类型，在二线治疗ORR、DCR、PFS和OS上均明显优于后者。接受二线化疗患者，其ORR为39.5%，DCR为59.2%，中位PFS和中位OS分别为3.3个月和5.3个月。据方案将二线化疗患者分组，敏感型复发患者由采用含VP-16方案的A组和采用含CPT-11方案的B1组组成，两组ORR分别为48.6%和35.3%，DCR分别为68.6%和58.8%，均无明显差异（P值分别为0.264和0.400）；两组二线中位PFS分别为4.0个月和3.0个月，无明显差异（*P*=0.432）；两组中位OS分别为6.5个月和4.5个月，无统计学差异（*P*=0.508）。耐药/难治型复发患者由其余含CPT-11方案的B2组、含PTX/DXL方案的C组和含TPT方案的D组组成。组间ORR、DCR、二线中位PFS无明显统计学差异（*P*值分别为0.521、0.528和0.775）；D组中位OS优于B2组和C组，差异具有统计学意义（P值分别为0.043、0.030）。四个方案组毒副作用相似，Ⅲ度-Ⅳ度血液学毒性组间并无差异；伊立替康组的患者腹泻发生率高于其他三组（*P*=0.029）。

**结论:**

二线化疗可以给一线治疗失败的小细胞肺癌患者带来生存获益；不同二线化疗方案患者的近期缓解和无进展生存相似；耐药/难治型患者二线化疗采用含TPT的方案可能会给患者带来更好的总生存获益。

## Abstract (en)

**Background and objective:**

Small-cell lung cancer (SCLC) is an aggressive disease for which the mainstay of treatment is cytotoxic chemotherapy. Despite good initial responses most patients will relapse or progress after the first-line therapy. The evidence of a benefit from second-line chemotherapy is limited in patients with relapsed/advanced SCLC. Some drugs are recommended by guidelines, but more regimens are formulated based on experience in clinical. So we conducted this retrospective study in order to compare the efficacy and safety of different second-line treatment regimens.

**Methods:**

We totally analyzed 309 patients received second-line treatment in our retrospective study. 157 patients received best supportive care (BSC), and the rest 152 patients received second-line chemotherapy. The *Kaplan*-*Meier* method survival curves and *Log*-*rank* test were used to analysis the differences among different groups. The endpoints were objective response rate (ORR), disease control rate (DCR), progression-free survival (PFS), and overall survival (OS).

**Results:**

Patients administered second-line chemotherapy lived significantly longer, with a total OS from first-line therapy of 11.5 mo compared to 6.0 mo in patients with best supportive care alone (*P* < 0.001), and the ORR, DCR, PFS and OS of the former (including the sensitive disease and resistance/refractory disease patients) were obviously better than that of the latter. The ORR and DCR of the patients who received second-line chemotherapy is 39.5% and 59.2%, respectively. The median PFS and OS from second-line chemotherapy were 3.3 mo and 5.3 mo. The patients who received second-line chemotherapy were divided by types of second-line regimens. The sensitive disease patients were from group A (VP-16-based rechallenge) and group B1 (CPT-11-based regimen). The ORR of the two groups were 48.6% and 35.3%, and the DCR were 68.6% and 58.8%, respectively. There was no statistically significant difference (*P*=0.264; *P*=0.400). The median PFS from second-line chemotherapy of the two groups were 4.0 mo and 3.0 mo, and the second-line median OS were 6.5 mo and 4.5 mo. There was no statistic difference (*P*=0.432; *P*=0.508). The resistance/refractory disease patients were divided into group B2 (CPT-11-based regimen), group C (PTX/DXL-based regimen) and group D (TPT-based regimen). There was no statistic difference in second-line ORR, DCR and median PFS among the three groups (*P* value is 0.521, 0.528 and 0.775, respectively); The median OS from second-line chemotherapy of the group D is longer than that of group B2 and group C, with statistical difference (*P*=0.043; *P*=0.030). The differences of grade Ⅲ-Ⅳ hematologic toxicities among the four subgroups were not statistically different. The incidence of diarrhea in non-hematologic toxicities in patients who received irinotecan as second-line chemotherapy was higher than other three subgroups (*P*=0.029).

**Conclusion:**

Patients who progressed after the completion of first-line chemotherapy can gain survival benefit. The response and the PFS of the different second-line chemotherapies were similar. The patients who received the TPT-based regimen may gain longer overall survival than other resistance/refractory disease patients.

小细胞肺癌（small cell lung cancer, SCLC）占所有肺癌的10%-15%，倍增速度快，极易出现早期远处转移和获得性耐药，是一种恶性程度极高的肿瘤^[1]^。SCLC一线化疗缓解率（response rate, RR）较高，局限期SCLC可达70%-90%，广泛期SCLC可达50%-60%^[2]^，但约80%的局限期患者和几乎所有的广泛期患者会在初始治疗1年内复发或进展^[3-5]^。目前针对SCLC二线化疗的有效性和毒性的研究较少。拓泊替康（topotecan, TPT）是目前唯一一个在欧洲和美国获准用于二线治疗SCLC的药物，而氨柔比星（amrubicin, AMR）、伊立替康（irinotecan, CPT-11）、紫杉醇（paclitaxel, PTX）、多西他赛（docetaxel, DXL）等其他药物在一些临床研究中，也表现出对复发的SCLC具有一定的治疗效果^[6-11]^，但目前对于复发或进展的SCLC尚无标准的二线治疗方案和大样本前瞻性试验结果以供参考，临床多依据经验制定方案，因此本回顾性研究的目的，旨在通过分析SCLC患者二线治疗的近期疗效、毒副反应、远期生存差异，探讨SCLC二线化疗的必要性及可选方案孰优孰劣，期望为临床治疗SCLC提供参考依据。

## 1 资料和方法

### 1.1 研究对象

选取2010年1月-2013年12月于解放军第307医院肺部肿瘤内科住院诊治的SCLC患者。所有患者需符合以下条件：①均经细胞学或组织病理学确诊为SCLC；②年龄大于18周岁；③在治疗前经过了系统的分期检查，根据美国退伍军人肺癌研究组（Veterans Administration Lung Study Group, VALG）的标准分为局限期（limited disease, LD）与广泛期（extensive disease, ED）SCLC患者^[12]^；④所有患者均接受过标准的一线化疗，在一线治疗失败后接受二线化疗或最佳支持治疗。

收集患者详细临床资料、主要的治疗相关毒性、随访时间及生存时间[总生存时间（overall survival, OS）、无进展生存时间（progression-free survival, PFS）]、疗效[完全缓解（complete response, CR）、部分缓解（partial response, PR）、稳定（stable disease, SD）、进展（progressive disease, PD）]。

### 1.2 治疗方案

对于敏感型复发的患者，二线治疗采用原一线方案或含CPT-11的方案；对于耐药/难治型复发的患者，二线治疗是不同于一线的新方案。一线方案均为顺铂或卡铂联合依托泊苷（Etoposide, VP-16）。二线方案分为4组：A组：含VP-16方案；B组：含CPT-11方案；C组：含PTX/DXL方案；D组：含TPT方案。四组中均有部分患者采用单药，部分患者采用联合卡铂或顺铂的双药方案。具体剂量：VP-16 60 mg/m^2^，静脉滴注，d1-d5；CPT-11 60 mg/m^2^-70 mg/m^2^，静脉滴注，d1，d8；PTX 150 mg/m^2^-175 mg/m^2^，静滴，d1；DXL 60 mg/m^2^-75 mg/m^2^，静滴，d1；TPT 0.75 mg/m^2^-1.2 mg/m^2^，d1-d5；。若联合顺铂：顺铂75 mg /m^2^，静滴，d1（或分3天给药）；若联合卡铂：卡铂曲线下面积（area under the curve, AUC）=5-6，静滴，d1（或分3天给药）。BSC组患者可接受除特异性抗肿瘤药物以外的治疗方法，以最大限度的提高生活质量，包括营养支持、镇痛、止吐、输血、胸膜腔穿刺及为缓解症状所进行的局部姑息放射^[13]^。

化疗前常规预防呕吐，化疗后出现的呕吐、血象异常及肝肾功异常等不良反应均按常规处理。本研究允许由于毒性引起的药物中断或剂量调整。

### 1.3 分析方法

收集患者完整的临床资料；体力评分采用美国东部肿瘤协作组体力评分（Eastern Cooperative Oncology Group Performance Status, ECOG PS）；所有患者均有影像学可评估的靶病灶，疗效评价依据实体肿瘤疗效评价标准1.1版（Response Evaluation Criteria in Solid Tumors, RECIST version 1.1）进行评估；不良反应按照1999年世界卫生组织（World Health Organization, WHO）抗癌药物毒性反应评定标准评价，分为0度-Ⅳ度；OS指患者开始治疗至死亡的时间（月）；PFS指患者开始治疗至出现疾病进展的时间（月）。

主要研究终点为二线化疗的PFS、OS，次要研究终点为客观缓解率（objective response rate, ORR）、疾病控制率（disease control rate, DCR）和安全性分析。

### 1.4 统计学方法

采用SPSS 19.0统计软件进行数据统计分析。定性资料采用卡方检验或*Fisher*精确检验，采用*Kaplan*-*Meier*法模拟患者OS及PFS，同时绘制生存曲线。通过*Kaplan*-*Meier*分析与*Log*-*rank*检验，以揭示组间差异。*P* < 0.05为差异有统计学意义。

## 2 结果

### 2.1 一线治疗

#### 2.1.1 患者特征

309例SCLC患者均接受一线铂类联合依托泊苷化疗后出现复发或进展，其中152例进展后给予了二线方案化疗，其余157例进展后仅给予最佳支持治疗（best supportive care, BSC）。一线治疗时患者年龄为20岁-81岁，中位年龄为57岁。男性占69%（213例），广泛期患者占86%（266例），ECOG PS 0-1分的患者占77%（238例），ECOG PS 2-3分的患者占23%（71例）。

#### 2.1.2 一线治疗疗效

所有的入组患者均接受了铂类联合依托泊苷的一线方案化疗，其中207例（67%）接受了顺铂（cisplatin）联合依托泊苷化疗，其余103例（33%）接受了卡铂（carboplatin）联合依托泊苷化疗。一线化疗中位周期数为5.0，ORR为79%，中位PFS为6.5个月，中位OS为8.3个月。

### 2.2 二线治疗

#### 2.2.1 患者特征

患者一线治疗出现进展后，其中152例（49%）接受了二线化疗，其余157例（51%）仅接受了BSC。两组患者年龄结构和一线化疗中位PFS存在差异（分别为5.5个月和5.0个月，*P*=0.043），其余临床病理特征无差异。患者特征见表 1。

**1:** 二线化疗组与二线最佳支持治疗组患者特征 Clinical characteristics of patients who received second-line therapy with chemotherapy or BSC

Clinical characteristics	Second-line therapy		*P*
	Chemotherapy (*n*=152)	BSC (*n*=157)	
Age (yr)			< 0.001
≤60	98 (64.5%)	64 (40.8%)	
> 60	54 (35.5%)	93 (59.2%)	
Gender			0.248
Male	109 (71.7%)	103 (65.6%)	
Female	43 (28.3%)	54 (34.4%)	
Smoking			0.925
Smoker	97 (63.8%)	101 (64.3%)	
Nonsmoker	55 (36.2%)	56 (35.7%)	
Stage at diagnosis			0.322
Limited disease	23 (15.1%)	19 (12.1%)	
Extensive disease	129 (84.9%)	138 (87.9%)	
First-line chemotherapy			0.354
Cisplatin+etoposide	100 (65.8%)	111(70.7%)	
Carboplatin+etoposide	52 (34.2%)	46 (29.3%)	
Radiotherapy			0.551
Yes	127 (83.6%)	135 (86.0%)	
No	25 (16.4%)	22 (14.0%)	
ECOG PS at diagnosis			0.135
0-1	117 (77.0%)	109 (69.4%)	
2-3	35 (23.0%)	48 (30.6%)	
ECOG PS at second-line			0.081
0-1	65 (14.4%)	52 (33.1%)	
2-3	87 (85.6%)	105 (66.9%)	
BSC: best supportive care; ECOG PS: Eastern Cooperative Oncology Group performance status; PFS: progression free survival.			

#### 2.2.2 疗效和生存分析

二线化疗和二线BSC患者ORR分别为39.5%和5.1%，差异有统计学意义（*P* < 0.001）。两组患者DCR分别为59.2%和8.9%，差异有统计学意义（*P* < 0.001）。二线化疗组和二线BSC组自诊断开始的总OS分别为11.5个月和6.0个月，通过*Kaplan*-*Meier*法绘制生存曲线，二线化疗组患者较二线最佳支持治疗组患者有明显的总生存获益（*P* < 0.001）。生存曲线见图 1。进一步分层分析，将二线采取化疗的患者按敏感型复发和耐药/难治型复发分组，分别与二线采取BSC患者比较。敏感型复发患者二线化疗ORR和DCR分别为42.0%和63.8%，耐药/难治型复发患者二线化疗ORR和DCR分别为33.7%和56.6%，均明显优于二线BSC患者（*P*值均小于0.001）；敏感型复发患者二线化疗PFS和OS分别为3.7个月和5.8个月；耐药/难治型患者二线化疗PFS和OS分别为3.0个月和5.0个月；二线BSC患者二线PFS和OS分别为2.0个月和3.7个月。经统计分析，二线采取化疗的敏感型复发患者或耐药/难治型复发患者，较二线采取BSC的患者，在二线治疗PFS、OS上均有明显获益（*P*值均小于0.001）。

**1:**
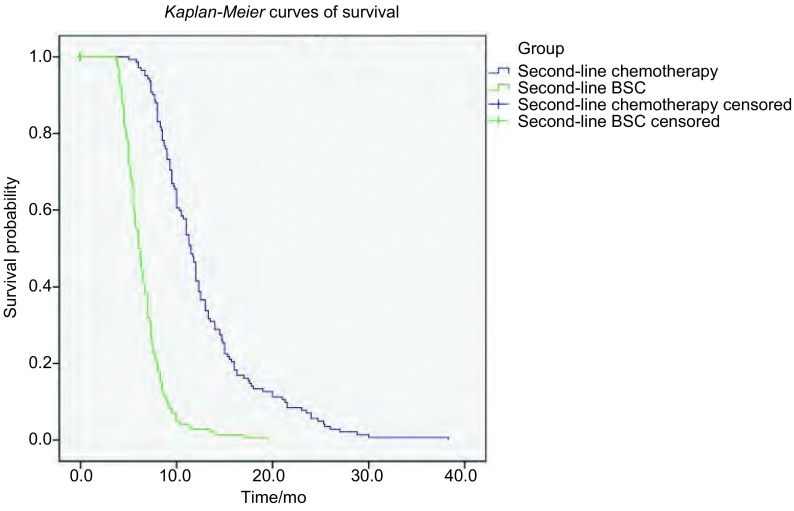
二线化疗组与二线最佳支持治疗组总生存曲线 *Kaplan*-*Meier* curves of overall survival in second-line chemotherapy group and second-line best supportive care group

### 2.3 二线化疗方案的疗效分析

#### 2.3.1 二线化疗患者特征

152例二线接受化疗的患者，根据方案不同将其分为四组，A组35例（23%）采用含依托泊苷方案；B组56例（37%）采用含CPT-11的方案；C组32例（21%）采用含PTX/DXL的方案；D组29例（19%）采用含TPT的方案。各方案组间在年龄、性别、吸烟史、诊断时的分期、体力评分、一线方案、化疗周期数、是否联合铂类等组成上具有可比性，但A组患者一线中位PFS明显长于其他三组（*P* < 0.001）。二线化疗各组患者特征见表 2。

**2:** 二线化疗各方案组患者临床特征 Clinical characteristics of patients who received different second-line chemotherapy regimens

Clinical characteristics	Second-line chemotherapy therapy				*P*
	Group A (*n*=35)	Group B (*n*=56)	Group C (*n*=32)	Group D (*n*=29)	
Age (yr)					0.985
≤60	23 (65.7%)	35 (62.5%)	21 (65.6%)	19 (37.9%)	
> 60	12 (34.3%)	21 (37.5%)	11 (34.4%)	10 (62.1%)	
Gender					0.723
Male	24 (68.6%)	38 (67.8%)	23 (71.9%)	17 (58.6%)	
Female	11 (31.4%)	18 (32.2%)	9 (28.1%)	12 (41.4%)	
Smoking					0.290
Smoker	22 (62.9%)	36 (64.3%)	21 (65.6%)	13 (79.3%)	
Nonsmoker	13 (37.1%)	20 (35.7%)	11 (34.4%)	16 (20.7%)	
Stage at diagnosis					0.557
Limited disease	7 (20.0%)	8 (14.3%)	3 (9.3%)	6 (20.7%)	
Extensive disease	28 (80.0%)	48 (85.7%)	29 (90.7%)	23 (79.3%)	
First-line chemotherapy					0.983
Cisplatin+etoposide	21 (60.0%)	35 (62.5%)	20 (62.5%)	17 (58.6%)	
Carboplatin+etoposide	14 (40.0%)	21 (37.5%)	12 (37.5%)	12 (41.4%)	
ECOG PS at diagnosis					0.722
0-1	27 (77.1%)	40 (71.4%)	26 (81.4%)	23 (79.3%)	
2-3	8 (22.9%)	16 (28.6%)	6 (18.6%)	6 (20.7%)	
Median PFS at 1st-line (mo)	8.0	5.0	4.5	5.5	< 0.001
ECOG PS at 2nd-line					0.338
0-1	22 (62.9%)	25 (44.6%)	15 (46.9%)	13 (44.8%)	
2-3	13 (37.1%)	31 (55.4%)	17 (53.1%)	16 (55.2%)	
No. of courses at 2nd-line					0.870
≤4	15 (42.9%)	26 (46.4%)	16 (50.0%)	14 (48.3%)	
5-6	20 (57.1%)	30 (53.6%)	16 (50.0%)	15 (51.7%)	
With platinum at 2nd-line					0.141
Yes	29 (82.9%)	51 (91.1%)	28 (87.5%)	21 (72.4%)	
No	6 (17.1%)	5 (8.9%)	4 (12.5%)	8 (27.6%)	
Group A: VP-16-based rechallenge; Group B: CPT-11-based regimen; Group C: PTX/DXL-based regimen; Group D: TPT-based regimen.					

#### 2.3.2 疗效和生存分析

所有二线化疗患者其二线化疗中位PFS为3.3个月，二线化疗中位OS为5.3个月。对于不同二线方案的疗效和生存分析，为规避复发类型不同所带来的影响，本研究将所有二线化疗患者分为敏感型复发和耐药/难治型复发分别分析（表 3）。敏感型复发患者由A组和部分B组（设为B1组）患者组成，耐药/难治型复发患者由C组、D组和其余部分B组（设为B2组）患者组成。69例敏感型复发患者中，35例采用含VP-16二线方案的患者和34例采用含CPT-11方案的患者，两组ORR分别为48.6%和35.3%，DCR分别为68.6%和58.8%，均无明显差异（*P*值分别为0.264和0.400）；两组二线中位PFS分别为4.0个月和3.0个月，无明显差异（*P*=0.432），生存曲线见图 2A；两组中位OS分别为6.5个月和4.5个月，无统计学差异（*P*=0.508），生存曲线见图 2B。83例耐药/难治型复发患者包括B2组、C组和D组，二线ORR分别为27.3%、31.3%和41.1%，DCR分别为50.0%、56.3%和62.1%，均无明显统计学差异（*P*值分别为0.533和0.689）；三组患者的中位PFS分别为2.6个月、3.0个月和3.0个月，无统计学差异（*P*=0.775），进一步两两比较，三组中位PFS无差异。三组的中位OS分别为3.8个月、4.5个月和5.3个月，无明显统计学差异（*P*=0.056），详见表 3。进一步两两比较发现，D组中位OS优于B2组和C组，差异具有统计学意义（*P*值分别为0.043、0.030），而B2组与C组中位OS比较无明显差异（*P*=0.608），生存曲线见图 3。

**3:** 不同复发类型小细胞肺癌患者不同二线方案的ORR、DCR、PFS、OS ORR, DCR, Median PFS, Median OS in months for sensitive disease patients or resistance/refractory disease patients after different second-line regimens

Item	Sensitive disease (*n*=69)				Resistance/refractory disease (*n*=83)			
	Group A (*n*=35)	Group B1 (*n*=34)	*P*		Group B2 (*n*=22)	Group C (*n*=32)	Group D (*n*=29)	*P*
CR	1 (2.9%)	2 (5.9%)	-		0 (0%)	0 (0%)	1 (3.4%)	-
PR	16 (45.7%)	10 (29.4%)	-		6 (27.3%)	10 (31.3%)	11 (37.9%)	-
SD	7 (20.0%)	8 (23.5%)	-		5 (22.7%)	8 (25.0%)	6 (20.7%)	-
PD	11 (31.4%)	14 (41.2%)	-		11 (50.0%)	14 (43.8%)	11 (37.9%)	-
ORR	48.6%	35.3%	0.264		27.3%	31.3%	41.4%	0.533
DCR	68.6%	58.8%	0.400		50.0%	56.3%	62.1%	0.689
PFS (mo)	4.0	3.0	0.432		2.6	3.0	3.0	0.775
OS (mo)	6.5	4.5	0.508		3.8	4.5	5.3	0.056
CR: complete response; PR: partial response; SD: stable disease; PD: progressive disease; ORR: objective response rate; DCR: disease control rate; OS: overall survival.								

**2:**
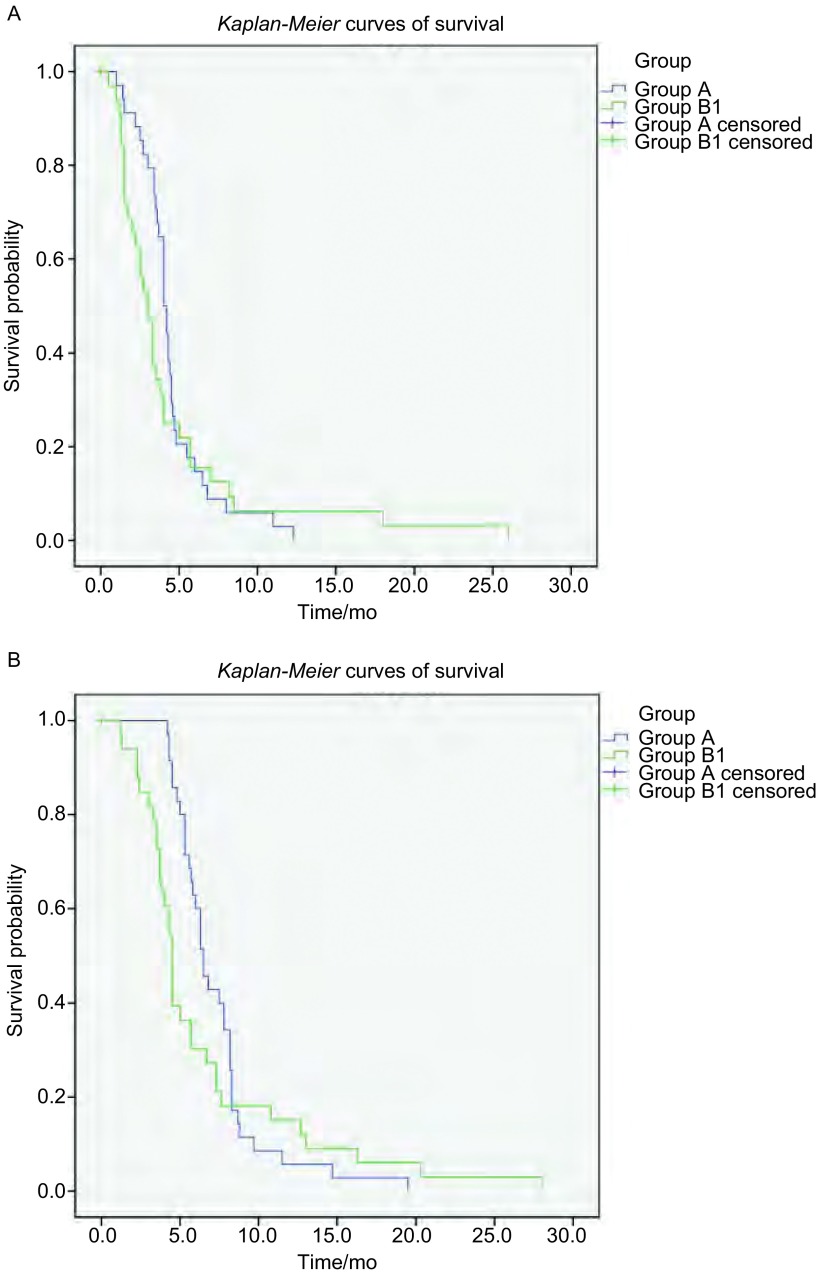
SCLC敏感复发患者二线不同化疗方案生存曲线。A：PFS比较；B：OS比较 *Kaplan*-*Meier* curves of survival in different second-line chemotherapy regimens groups of SCLC sensitive disease patients. A: *Kaplan*-*Meier* curves of progression-free survival (PFS); B: *Kaplan*-*Meier* curves of overall survival (OS)

**3:**
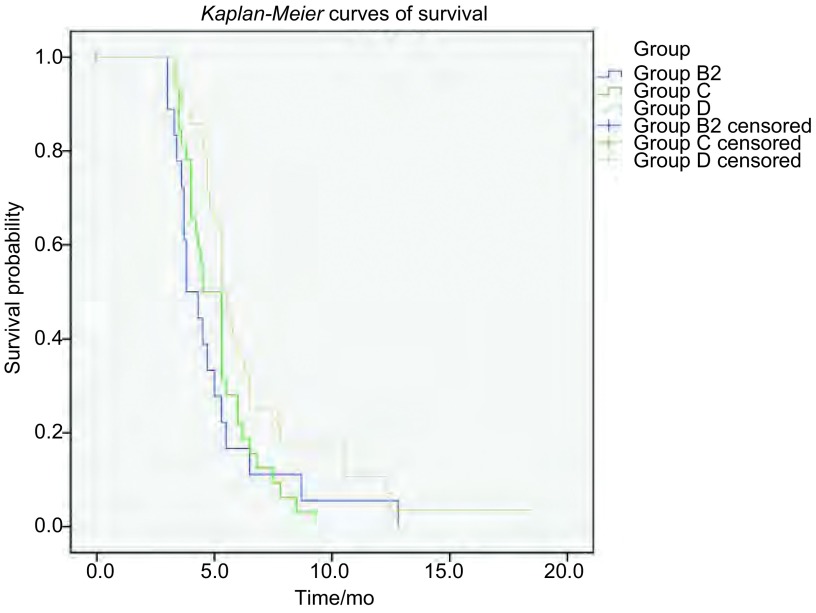
SCLC耐药/难治复发患者二线不同化疗方案OS曲线 *Kaplan*-*Meier* curves of OS in different second-line chemotherapy regimens groups of SCLC resistance/refractory disease patients

同时我们将二线化疗的近期疗效同一线化疗的近期疗效做了相关性分析，发现两者呈正相关（*P*=0.001），一线疗效较好的患者在进展后获得较好的二线化疗反应的可能性更大（表 4）。

**4:** 小细胞肺癌患者二线化疗的反应与一线化疗的关系 Correlation between response to first- and second-line chemotherapy

Response to first-line	Response to second-line				
	CR	PR	SD	PD	Not evaluable
CR	2 (13.3%)	6 (40.0%)	5 (33.3%)	1 (6.7%)	1 (6.7%)
PR	0 (0)	49 (53.8%)	35 (38.5%)	5 (5.5%)	2 (2.2%)
SD	0 (0)	1 (3.7%)	13 (48.1%)	12 (44.4%)	1 (3.7%)
PD	0 (0)	2 (10.5%)	6 (31.6%)	11 (57.9%)	0 (0)

#### 2.3.3 安全性分析

所有二线化疗患者未出现治疗相关性死亡事件。121例（79.6%）二线化疗患者在治疗过程中出现毒副反应，其中最常见的血液学毒性为白细胞减少症和贫血，Ⅲ度-Ⅳ度白细胞减少症和贫血发生率分别为32.4%和22.6%，Ⅲ度-Ⅳ度血小板减少的发生率为17.7%，四组患者Ⅲ度-Ⅳ度血液学毒性并无差异（表 5）。最常见的非血液学毒性为乏力，发生率为59.2%；其次为胃肠道反应（包括呕吐、恶心、腹泻），发生率为41.2%。含CPT-11方案组的患者，其Ⅲ度-Ⅳ度腹泻发生率高于其他三组，差异具有统计学意义（*P*=0.029）。其他的血液学毒性及非血液学毒性发生率较低，且较易耐受（表 5）。

**5:** 不同二线方案组间的Ⅲ度-Ⅳ度不良反应 Grade Ⅲ-Ⅳ toxicities in 152 patients receiving second-line chemotherapy

Toxicity	Group A	Group B	Group C	Group D	*P*
Hematologic					
Leukopenia	12 (34.3%)	23 (41.1%)	15 (46.9%)	9 (31.0%)	0.565
Anemia	9 (25.7%)	14 (25.0%)	6 (18.8%)	5 (17.2%)	0.770
Thrombocytopenia	7 (20.0%)	12 (21.4%)	5 (15.6%)	3 (10.3%)	0.609
Non-hematologic					
Fatigue	18 (51.4%)	22 (39.2%)	13 (40.6%)	10 (34.5%)	0.545
Nausea or vomiting	14 (40.0%)	18 (32.1%)	9 (28.1%)	7 (24.1%)	0.557
Diarrhea	9 (25.7%)	25 (44.6%)	5 (15.6%)	8 (27.6%)	0.029
ALT increased	4 (11.4%)	8 (14.3%)	4 (12.5%)	3 (10.3%)	0.956
AST increased	4 (11.4%)	7 (12.5%)	3 (9.4%)	4 (13.8%)	0.956
ALT: alanine transaminase; AST: aspartate transaminase.					

### 2.4 后续治疗

二线化疗进展后有52例患者（34.2%）接受了三线化疗。这其中接受四线以及四线以上治疗的患者仅有12例（7.9%）。接受了三线以上化疗的这部分患者，其二线化疗的ORR为38%，其二线化疗的中位PFS、中位OS分别为3.0个月和5.5个月，与接受二线化疗的所有患者无统计学差异。

## 3 讨论

美国国家癌症网络（National Comprehensive Cancer Network, NCCN）小细胞肺癌指南（2014.V2）对于复发或晚期的SCLC二线治疗，推荐包括托泊替康、紫杉醇、多西紫杉醇、伊立替康、长春瑞滨、吉西他滨、异环磷酰胺、替莫唑胺、依托泊苷口服剂型等多种联合或单药化疗，但仅推荐托泊替康为1类证据，这是基于两项Ⅲ期临床试验得出的^[13, 14]^。但是对于其他的二线化疗药物，目前尚无足够证据证明其临床获益超过拓泊替康。

本研究主要目的为通过对真实临床实践中SCLC病例的回顾性分析，探讨经一线标准方案治疗后出现复发或进展的SCLC患者的二线治疗情况，从而为临床治疗提供参考。主要研究终点为二线化疗的PFS、OS，次要研究终点为ORR、DCR和安全性分析。

我们首先比较二线采取化疗和BSC的患者之间的生存差异，结果表明SCLC患者一线治疗失败后，采取二线化疗可以给患者带来生存获益（median OS: 11.5 months *vs* 6.0 months; *P* < 0.001），并且二线接受化疗的患者，其ORR和DCR均明显高于二线仅接受最佳支持治疗的患者，同时分层分析表明，无论是敏感型复发还是耐药/难治型复发患者，二线采取化疗较二线BSC治疗均有PFS、OS获益，说明无论对一线标准方案原发耐药或是继发耐药，二线采取推荐方案化疗，较二线采取BSC治疗会给患者带来生存获益，这与以往的相关研究^[16]^类似。

对于二线采取化疗的患者，我们分析了其PFS、OS、ORR和DCR。这部分患者二线化疗总体的ORR为39.5%，总体DCR为59.2%，符合文献报道的7%-73%^[17-19]^，而且二线化疗的ORR、DCR明显低于一线化疗^[20]^。同时对不同二线方案的ORR、DCR进行分析，发现无论是敏感型复发或者是耐药/难治型复发，各方案间的二线ORR、DCR并无统计学差异，说明指南推荐的SCLC二线不同方案间无近期缓解率和疾病控制率差异。Huisman^[21]^和Glisson^[22]^等曾报道了SCLC一线未曾获益的患者，其二线获益的可能也较低，我们也对一线、二线治疗的近期疗效进行相关性分析，提示二线化疗的疗效与一线化疗的疗效相关，一线化疗疗效较好的患者，二线化疗获得客观缓解的可能性更大。

本研究所有接受二线化疗患者的二线PFS和OS分别为3.3个月和5.3个月，这与国外已发表的文献^[23, 24]^报道接近。如不考虑患者复发类型，A组患者因为对一线方案更敏感，一线治疗后缓解时间较长，存在更高比例的化疗敏感肿瘤细胞^[25, 26]^，并且这部分患者二线治疗前经过了较长的恢复期，体力状况较好，也可能与这部分患者的后续多线化疗有更多的选择方案^[22]^，所以其在二线化疗生存期上存在优势。所以我们将二线化疗患者分为敏感型复发和耐药/难治型复发两部分分别分析（表 3）。结果提示对于敏感型复发患者，二线采用一线原方案的A组，与换用含CPT-11方案的B1组，在二线中位PFS和中位OS上均无明显统计学差异（*P*值分别为0.432和0.508）；而对于耐药/难治型复发患者，分别采用含CPT-11、含PTX/DXL、含TPT的B2组、C组、D组，在二线中位PFS分别为2.6个月、3.0个月和3.0个月，无统计学差异（*P*=0.775）。三组的中位OS分别为3.8个月、4.5个月和5.3个月，无统计学差异（*P*=0.056）；进一步两两比较发现，含TPT的D组中位OS优于含CPT-11的B2组和含PTX/DXL的C组，差异有统计学意义（*P*值分别为0.043、0.030），而B2组与C组中位OS比较无明显差异（*P*=0.608）。二线不同方案对进展或复发后的SCLC的疾病进展控制时间接近，不同于一线原方案的新的方案虽然不存在继发性耐药，但并没有带来PFS获益，说明二线方案原发耐药是导致有效率和疾病控制时间较短的主要原因。二线化疗的OS时间更能说明患者能否从二线治疗中获得生存获益。敏感型复发患者中，A组和B1组患者在二线中位OS上无明显差异，可能说明两种方案对于敏感型复发患者的二线治疗疗效接近，临床选择可针对患者一般状况、对毒副作用的耐受性等做出选择。而耐药/难治型复发患者中，二线采用含TPT方案的患者，在二线中位OS上较其他两种方案更能获益，可能因为该研究中患者临床使用TPT时剂量灵活度高，副反应易耐受，患者依从性较好，同时密切监测血液学毒性，及时给予支持治疗相关，也可能与患者病例数较少等回顾性分析带来的偏差相关，需要进一步的前瞻性试验，才能明确上述结论。

二线化疗最常见的血液学毒性为白细胞减少症和贫血，最常见的非血液学毒性为乏力。四个方案组患者二线化疗的Ⅲ度-Ⅳ度血液学毒性发生率接近；在胃肠道反应方面，采用含CPT-11组的患者的迟发型腹泻发生率高于其他三组，表明采用该方案时需密切关注对腹泻的控制。

本研究虽为单中心回顾性研究，但也有其优势，比如在患者的来源、治疗方案的实施和疗效评估、副反应处理等方面均存在较好的一致性。通过对不同二线治疗方案的ORR、DCR、PFS和OS，以及毒副反应等进行分析，表明二线化疗可以给一线治疗失败的SCLC患者带来生存获益，其在SCLC二线治疗中具有重要地位；并且初步提示SCLC二线化疗各方案疗效和生存获益相似，采用含TPT的方案可能会给患者带来更好的总生存获益。我们期待更多中心及更多患者纳入分析，以进一步验证研究结果，并进行多中心、前瞻性的科学研究来说明SCLC二线化疗的最佳方案选择。

## References

[1] Lally BE, Urbanic JJ, Blackstock AW (2007). Small cell lung cancer: have we made progress over the last 25 years?. Oncologist.

[2] Cheng S, Evans WK, Stys-Norman D (2007). Chemotherapy for relapsed small cell lung cancer: a systematic review and practice guideline. J Thorac Oncol.

[3] Hurwitz JL, McCoy F, Scullin P (2009). New advances in the second-line treatment of small cell lung cancer. Oncologist.

[4] Rodriguez E, Lilenbaum RC (2010). Small cell lung cancer: past, present, and future. Curr Oncol Rep.

[5] Fischer B, Arcaro A (2008). Current status of clinical trials for small cell lung cancer. Rev Recent Clin Trials.

[6] Ando M, Kobayashi K, Yoshimura A (2004). Weekly administration of irinotecan (CPT-11) plus cisplatin for refractory or relapsed small cell lung cancer. Lung Cancer.

[7] Groen H, Fokkema E, Biesma B (1999). Paclitaxel and carboplatin in the treatment of small-cell lung cancer patients resistant to cyclophosphamide, doxorubicin, and etoposide: a non-cross-resistant schedule. J Clin Oncol.

[8] Inoue A, Sugawara S, Yamazaki K (2008). Randomized phase Ⅱ trial comparing amrubicin with opotecan in patients with previously treated small-cell lung cancer: North Japan Lung Cancer Study Group Trial 0402. J Clin Oncol.

[9] Masuda N, Fukuoka M, Kusunoki Y (1992). CPT-11: a new derivative of camptothecin for the reatment of refractory or relapsed small-cell lung cancer. J Clin Oncol.

[10] Onoda S, Masuda N, Seto T (2006). Phase Ⅱ trial of amrubicin for treatment of refractory or relapsed small-cell lung cancer: Thoracic Oncology Research Group Study 0301. J Clin Oncol.

[11] Smit E, Fokkema E, Biesma B (1998). A phase Ⅱ study of paclitaxel in heavily pretreated patients with small-cell lung cancer. Br J Cancer.

[12] Postmus PE, Berendsen HH, van Zandwijk N (1987). Retreatment with the induction regimen in small cell lung cancer relapsing after an initial response to short term chemotherapy. Eur J Cancer Clin Oncol.

[13] Jassem J, Ramlau R, Santoro A (2008). Phase Ⅲ trial of pemetrexed plus best supportive care compared with best supportive care in previously treated patients with advanced malignant pleural mesothelioma. J Clin Oncol.

[14] von Pawel J, Schiller JH, Shepherd FA (1999). Topotecan versus cyclophosphamide, doxorubicin, and vincristine for the treatment of recurrent small-cell lung cancer. J Clin Oncol.

[15] O'Brien ME, Ciuleanu TE, Tsekov H (2006). Phase Ⅲ trial comparing supportive care alone with supportive care with oral topotecan in patients with relapsed small-cell lung cancer. J Clin Oncol.

[16] Planchard D, Le Péchoux C (2011). Small cell lung cancer: new clinical recommendations and current status of biomarker assessment. Eur J Cancer.

[17] Kallianos A, Rapti A, Zarogoulidis P (2013). Therapeutic procedure in small cell lung cancer. J Thorac Dis.

[18] Eckardt JR, von Pawel J, Pujol JL (2007). Phase Ⅲ study of oral compared with intravenous topotecan as second-line therapy in small-cell lung cancer. J Clin Oncol.

[19] Fukuoka M, Furuse K, Saijo N (1991). Randomized trial of cyclophosphamide, doxorubicin, and vincristine versus cisplatin and etoposide versus alternation of these regimens in small-cell lung cancer. J Natl Cancer Inst.

[20] Tiseo M, Ardizzoni A (2007). Current status of second-line treatment and novel therapies for small cell lung cancer. J Thorac Oncol.

[21] Huisman C, Postmus PE, Giaccone G (1999). Second-line chemotherapy and its evaluation in small cell lung cancer. Cancer Treat Rev.

[22] Glisson BS (2003). Recurrent small cell lung cancer: update. Semin Oncol.

[23] Sundstrom S, Bremnes RM, Kaasa S (2005). Second-line chemotherapy in recurrent small cell lung cancer. Results from a crossover schedule after primary treatment with cisplatin and etopiside (EP-regimen) or cyclophosphamide, epirubicin, and vincristin (CEV-regimen). Lung Cancer.

[24] Kim YH, Goto K, Yoh K (2008). Performance status and sensitivity to first-line chemotherapy are significant prognostic factors in patients with recurrent small cell lung cancer receiving second-line chemotherapy. Cancer.

[25] Simon GR, Wagner H (2003). Small cell lung cancer. Chest.

[26] Levy B, Saxena A, Schneider BJ (2013). Systemic therapy for small cell lung cancer. J Natl Compr Canc Netw.

